# Thermal Cycling Life Prediction of Sn-3.0Ag-0.5Cu Solder Joint Using Type-I Censored Data

**DOI:** 10.1155/2014/807693

**Published:** 2014-07-08

**Authors:** Jinhua Mi, Yan-Feng Li, Yuan-Jian Yang, Weiwen Peng, Hong-Zhong Huang

**Affiliations:** School of Mechanical, Electronic and Industrial Engineering, University of Electronic Science and Technology of China, No. 2006, Xiyuan Avenue, West Hi-Tech Zone, Chengdu, Sichuan 611731, China

## Abstract

Because solder joint interconnections are the weaknesses of microelectronic packaging, their reliability has great influence on the reliability of the entire packaging structure. Based on an accelerated life test the reliability assessment and life prediction of lead-free solder joints using Weibull distribution are investigated. The type-I interval censored lifetime data were collected from a thermal cycling test, which was implemented on microelectronic packaging with lead-free ball grid array (BGA) and fine-pitch ball grid array (FBGA) interconnection structures. The number of cycles to failure of lead-free solder joints is predicted by using a modified Engelmaier fatigue life model and a type-I censored data processing method. Then, the Pan model is employed to calculate the acceleration factor of this test. A comparison of life predictions between the proposed method and the ones calculated directly by Matlab and Minitab is conducted to demonstrate the practicability and effectiveness of the proposed method. At last, failure analysis and microstructure evolution of lead-free solders are carried out to provide useful guidance for the regular maintenance, replacement of substructure, and subsequent processing of electronic products.

## 1. Introduction

With the development of electronic devices, the high-integrity and portability as well as the layout design have become important features of modern electronic devices. However, they are also encountered with some challenges in the electronic industry. The potential random vibrations and thermal shocks directly affect the quality and reliability of electronic devices [1–3] during transportation process and daily usage. According to statistical analysis presented in [1], about 70 percent of the electronic product failures are packaging structural failures. Solder joints are used to transmit the electrical signal and also serve as structural support of microelectronic structures. As a weak point of the electronic packaging structures, the solder joints of electronic products often suffer from the joint effect of electricity, heat, and load. The main failures of the solder joints include thermally induced failures, mechanically induced failures, and electrochemically induced failures [2, 3].

With the increase of environmental protection awareness and the European Union (EU) legislation on restriction of the use of certain hazardous substances (RoHS) and waste electrical and electronic equipment (WEEE) [4] taken effect in July 2006, Pb solders have been gradually replaced by SnAgCu alloys which are lead-free solders. However, such materials have many limitations, such as low durability and frangibility, which emerged in the service, especially in the high strain-stress situations. Due to the coefficients of thermal expansion (CTE) mismatch among surface mount components, printed circuit boards (PCBs), and solders, the solder joints will suffer from periodic stress and strain. It can cause crack initiation and propagation in the solders and eventually lead to functional failure of entire packaging structure [5]. Over the past decades, lots of researchers have focused on improving the reliability of lead-free solders which suffer from a joint effect of load, heat, and electricity during their lifecycle [6–11]. The mechanical properties of the solders, such as mechanical fatigues, thermal fatigues, shock, and creep deformations, have raised many concerns [6–9]. There are lots of studies on electromigration of the solder joints under high current density and the mechanical properties of BGA solder joints [10, 11]. The reliability study of the solder joints possesses high academic value and good market prospect.

The fatigue life of a solder joint is a basic merit of its reliability. Several fatigue life models, including Coffin-Manson model, Engelmaier model, and Solomon model, have been proposed based on plastic strain of solders [12–14]. Darveaux presented a life prediction model with 4 correlation coefficients, which has been widely used in the solder joint life prediction [15]. There are some creep strain-based fatigue models, such as the Knecht and Fox model and the Syed model [16], which are used to predict the creep fatigue life of solder joints. When the effect of plastic and creep strain is considered, Miner's linear superposition thermo can be applied to combine the plastic strain-based model with the creep strain-based model, and it can also be used to calculate the number of cycles to failure of products [16, 17].

Generally, it is hard to obtain exact failure time of modern products, especially for electronic products with high reliability. We commonly get different kinds of censored data, such as fixed-time censoring data and fixed-number censoring data, which are separately called type-I and type-II censored data. For censored data, Chen et al. [18, 19] presented a maximum likelihood estimate (MLE) method based on several kinds of censored data with Log-normal distribution and Weibull distribution. Huang et al. [20] proposed a new Bayesian reliability analysis method for processing fuzzy lifetime data. Many works related to type-I and type-II censored data have been presented by Balakrishnan et al. [21, 22]. However, according to the literature review, there is no work related to the fatigue life prediction of lead-free solder joints with censored data gathered from accelerated life tests.

In this paper, a thermal cycling test of lead-free ball grid array (BGA) and fine-pitch ball grid array (FBGA) connection structures is conducted, and failure data are collected. A fatigue life model and a type-I interval censored data processing method are used to predict the cycles to failure of lead-free solder joints. In order to verify the practicability and effectiveness of the proposed method, the results obtained by the proposed method are compared with the ones obtained by Minitab statistical software which is based on the built-in MLE method with two-parameter Weibull distribution. The Matlab curve fitting toolbox is used to check whether the lifetime of solder joint follows the Weibull distribution.

The remainder of this paper is organized as follows. The thermal cycling tests are introduced in Section 2.  Section 3 briefly reviews the modified Engelmaier model and the analysis method for Type-I censored data. In Section 4, the failure and microstructure of solder interface are analyzed first. Then, the fatigue life of solder joints based on the theory introduced in Section 3 is obtained. A comparison of predicted lifetime between the modified Engelmaier model and Matlab numerical simulation is conducted. We then conclude the paper in Section 5.

## 2. Thermal Cycling Test

### 2.1. Device under Test

**Figure 1:**
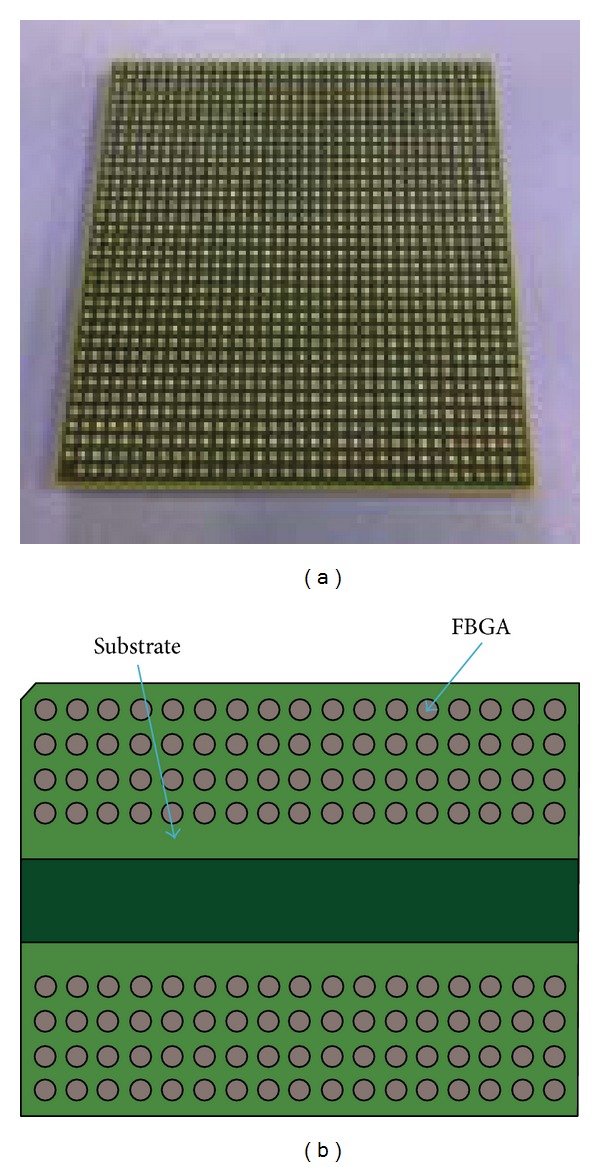
Package dimension of the BGA and the FBGA: (a) the BGA package and (b) the FBGA package.

**Figure 2:**
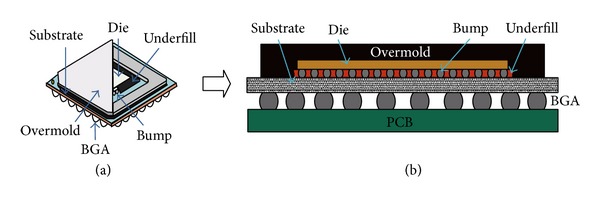
The detail package structure of the BGA (a) basic structure and (b) the details of the BGA.

This paper focuses on the life prediction of BGA and FBGA (Sn-3.0Ag-0.5Cu (wt.%)) packages.  Figure 1(a) depicts the bottom side of a BGA package. The perfect symmetrical distribution of the solder balls can be observed. The package dimension is 35 × 35 × 3.63 mm^3^ with an array of 34 × 34 Sn3.0Ag0.5Cu (SAC305) solder balls, where each solder ball has a diameter of 600 *μ*m. Ignoring the first mark point, there are 1155 solder balls, and each of them has a height of 0.5 ± 0.1 mm and 1 mm pitch. The package dimension of the FBGA is 14 × 10 × 1.1 mm^3^ as shown in Figure 1(b). There are 136 solder balls with an array of 8 × 17 symmetrical distribution. The height is 0.35 ± 0.05 mm and ball pitch is 0.8 mm.  Figure 2(a) shows the basic structure of the BGA package, and Figure 2(b) shows the detailed package structure of BGA interconnection structures.

The samples used in this paper were fabricated on printed circuit board assemblies (PCBAs) with qualified functionality, which have passed online testing, visual testing, and functional testing. Flame Retardant 4 (FR-4) substrates with Cu pads were used to assemble various components by SnAgCu solder pasted through a standard surface mounting technology (SMT) process. The *T*_*g*_ point (glass transition temperature) of FR-4 is from 130°C to 145°C, and it can withstand a temperature ranging from 260°C to 280°C, which makes it possible to meet the requirements of the lead-free SMT.

### 2.2. Design of Experiment (DOE)

**Figure 3:**
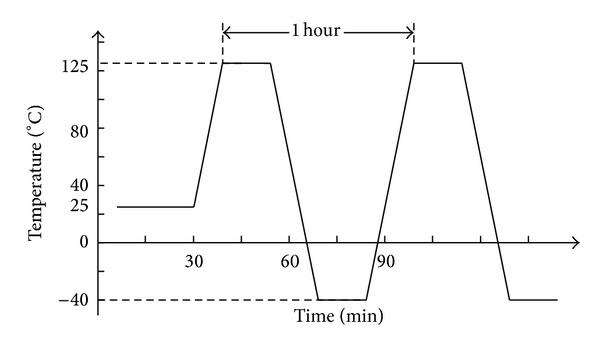
ATC thermal profile.

According to the JEDEC Standard of JESD22-A104C [23], an accelerated temperature cycling (ATC) test was conducted in a thermal chamber, where the temperature ranged from −40°C to 125°C. Each cycle lasted for 1 hour, including a 15-minute dwelling at −40°C and 125°C, respectively, and 15 minutes for ramping up and cooling down, as shown in Figure 3. The cycling began by ramping from an ambient temperature (25°C) to the highest temperature (125°C). Thus, for the analysis of cycling phase, the zero strain reference temperature was set to the ambient temperature. When the thermal cycling was completed at 250, 500, 750, 1000, 1500, 2000, and 2500 cycles for the BGA solder joints and 300, 600, 800, 1000, 1400, 1800, 2200, and 2500 cycles for the FBGA solder joints, 2 samples were taken out at each observation cycle. They are prepared by cross section, dye and pry, and standard metallographic procedures (grinding, polishing, and etching). Scanning electron microscope (SEM) was used to characterize the microstructures of the prepared solder matrices. At last, failure data were collected for reliability analysis, and failure analysis was performed on the assemblies.

## 3. Basic Theories of Fatigue Life Prediction

### 3.1. Fatigue Theory of Lead-Free Solder Joint Life Prediction

In the Coffin-Manson fatigue life model [13], the number of cycles to failure of solder joint (*N*_*f*_) can be expressed as an exponential relationship. It is established by the fatigue ductility coefficient (*ε*_*f*_′), the fatigue ductility exponent (*c*), and the plastic strain amplitude of each cycle (Δ*ε*_*p*_), which is given as follows:

(1) $$\begin{array}{c} \frac{\Delta \varepsilon_{p}}{2}=\varepsilon_{f}^{\prime }{\left(2N_{f}\right)}^{c}. \end{array}$$

Equation (1) is suitable for the case where the damage of solders completely depends on the plastic deformation. When some other factors, such as creep and plastic relaxation, are considered, the Coffin-Manson fatigue life model is modified as the Engelmaier fatigue model [14]. The number of cycles to failure of fatigue damage is determined by the total shear strain and the modified fatigue ductility index *c*. The fatigue life *N*_*f*_(*x*%) at a given failure probability *x* of surface mount solder attachment is given by [24]

(2) $$\begin{array}{c} N_{f}\left(x\%\right)=0.5{\left(\frac{\Delta \gamma }{2\varepsilon_{f}}\right)}^{1/c}{\left(\frac{\ln\left(1-0.01x\right)}{\ln0.5}\right)}^{1/m}, \end{array}$$

where Δ*γ* is the cyclic fatigue damage (the cyclic plastic strain range), *ε*_*f*_ is the fatigue ductility coefficient, and *m* is the Weibull shape parameter. The modification of the fatigue ductility exponent *c* takes the effect of the temperature *T*_*s*_ and the cycle frequency* f* into account, and *c* = −0.442 − (6 × 10^−4^)*T*_*s*_ + 1.74 × 10^−2^ln⁡⁡(1 + *f*). Considering the influence of the creep fatigue, the cyclic fatigue damage Δ*γ* used in (2) can be replaced by Δ*D* which is the cycle fatigue damage parameter and contains the creep damage and plastic relaxation [25]. When plastic and creep strain is the cause for solder joints fatigue crack, according to Miner's linear superposition theorem, we can get a plastic deformation and creep based fatigue life equation by combining the Knecht and Fox creep model with the Solomon fatigue model [16, 17].

When *x* = 50, we can get ln⁡(1 − 0.01*x*)/ln⁡0.5 = 1, and (2) can be simplified to [26]

(3) $$\begin{array}{c} N_{f}\left(50\%\right)=0.5{\left(\frac{\Delta \gamma }{2\varepsilon_{f}}\right)}^{1/c}. \end{array}$$

### 3.2. Type-I Censored Data Processing

In engineering practice, especially in storage reliability research, the following situation often happens. Suppose that the observation time points are denoted as *c*_1_,…, *c*_*n*_ and assume that the product fails at time *c*_*i*_, then the result is recorded as *δ*_*i*_ = 1; otherwise, it is recorded as *δ*_*i*_ = 0. The results *δ*_1_,…, *δ*_*n*_ can be expressed as follows [18]:

(4) $$\begin{array}{c} \delta_{i}=\{\begin{array}{cc} 1, & x_{i}\leq c_{i}\\ 0, & x_{i}>c_{i} \end{array}\;\left(i=1,\ldots ,n\right), \end{array}$$

where *x*_1_,…, *x*_*n*_ are the real fatigue life of *n* products. However, the values of *x*_1_,…, *x*_*n*_ may not be observed. We further assume that they are independently identically distributed. The Weibull distribution is used since it is one of the most commonly used distributions in reliability engineering. The distribution function of lifetime *T* is denoted as *F*(*t*, *θ*)    (*θ* ∈ Θ), and its probability density function is *f*(*t*, *θ*). Here Θ is a nonempty open set of *R* ^*m*^, which represents the set of distribution parameters. When the life of electronic product *T* follows a Weibull distribution, we have

(5) $$\begin{array}{c} P\left(T\leq t\right)=F\left(t;\eta ,m\right)=\{\begin{array}{cc} 1-\exp\left\{-{\left(\frac{t}{\eta }\right)}^{m}\right\}, & t>0,\\ 0, & t\leq 0, \end{array} \end{array}$$

where*η* is the scale parameter and* m* is the shape parameter with *η* > 0 and *m* > 0.

The probability density function of the two-parameter Weibull distribution is *f*(*t*) = (*m*/*η*)(*t*/*η*)^*m*−1^exp⁡{−(*t*/*η*)^*m*^}. The failure rate function is *λ*(*t*) = (*m*/*η*)(*t*/*η*)^*m*−1^, and the mean time to failure is MTTF = *η* · Γ(1 + 1/*m*) [27, 28]. The reliability of products at mission time *t*_0_ can be expressed as *R*(*t*_0_) = *P*(*T* > *t*_0_) = exp⁡{−(*t*/*η*)^*m*^}. The reliable life of the products having reliability of *R* can be obtained as *t*_*R*_ = *η*(−ln⁡*R*)^1/*m*^.

The product life distribution function is *F*_*s*_(*t*; *η*_*s*_, *m*_*s*_) in an actual working environment, and the lifetime *T* follows this distribution function in accelerated life test environment. The corresponding likelihood function of the observed data *δ*_1_,…, *δ*_*n*_ can be expressed as follows [18, 19]:

(6) L(η,m)=∏i=1n[F′(ci;η,m)]δi[1−F(ci;η,m)]1−δi=∏i=1n[1−exp⁡{−(ciη)m}]δi[exp⁡{−(ciη)m}]1−δi.

And the log-likelihood function is given as follows:

(7) ln⁡L=ln⁡{∏i=1n[1−exp⁡{−(ciη)m}]δi[exp⁡{−(ciη)m}]1−δi}=l(λ,m)=∑i=1n[δiln⁡(1−exp⁡{−(ciη)m})−(1−δi)λcim],

where *λ* = *η* ^−*m*^. The derivative of *λ* is given as follows:

(8) $$\begin{array}{c} \frac{\partial l}{\partial \lambda }=\overset{n}{\underset{i=1}{\sum }}c_{i}^{m}-\overset{n}{\underset{i=1}{\sum }}\frac{\delta_{i}c_{i}^{m}}{1-\exp\left\{-{\left(c_{i}/\eta \right)}^{m}\right\}}. \end{array}$$

If the range of *m* can be obtained as *m* ∈ [*m*_1_, *m*_2_] and 0 < ∑_*i*=1_^*n*^ *δ*_*i*_ < *n*, then ∂*l*/∂*λ* is a strict continuous increasing function. And if $\hat{\lambda }=\tilde{\lambda }(\hat{m})$, $\hat{\lambda }$ and $\hat{m}$ are the maximum likelihood estimation of *λ*, *m*, respectively.

When 0 < ∑_*i*=1_^*n*^ *δ*_*i*_ < *n*, the following method can be used to obtain $\hat{\lambda }$ and $\hat{m}$. If *m*_1_ = *m* ^(1)^ < *m* ^(2)^ < ⋯<*m* ^(*l*)^ = *m*_2_ and *m* ^(*i*+1)^ − *m* ^(*i*)^ are extremely small, the dichotomy method [18] can be used to solve the following formula at each *m* ^(*k*)^:

(9) $$\begin{array}{c} \overset{n}{\underset{i=1}{\sum }}\frac{\delta_{i}c_{i}^{m^{\left(k\right)}}}{1-\exp\left\{-\lambda c_{i}^{m^{\left(k\right)}}\right\}}=\overset{n}{\underset{i=1}{\sum }}c_{i}^{m^{\left(k\right)}}. \end{array}$$

When all *λ* ^(*k*)^ are obtained, *k*_0_ satisfies *l*(*λ* ^(*k*_0_)^, *m* ^(*k*_0_)^) = max⁡{*l*(*λ* ^(*k*)^, *m* ^(*k*)^) : *k* = 1,2,…, *l*}. Then, the corresponding *λ* ^(*k*_*o*_)^ and *m* ^(*k*_0_)^ can be determined.

### 3.3. Acceleration Factor Modeling

In order to extrapolate the normal life characteristics, the life characteristics under a high stress should be used. There are four commonly used acceleration models, that is, the Arrhenius model, the inverse power law model, the Eyring model, and the temperature-humidity model. The acceleration factor (AF) is an important parameter used in the accelerated life testing. It is the ratio from the normal stress life characteristics to the life characteristics under a high stress level.

An extension has been developed by Norris and Landzberg [29]. The computation of the acceleration factor AF_NL_ for SnPb eutectic can be expressed as [30]

(10) AFNL=N0Nt=(ΔTtΔT0)1.9(f0ft)0.333×exp⁡{1414(1Tmax⁡,0−1Tmax⁡,t)}.

The calculation of the AF depends on the temperature difference between the Δ*T*_0_ and the Δ*T*_*t*_, the cycle frequencies *f*_0_ and *f*_*t*_, and the maximum temperatures *T*_max⁡,0_ and *T*_max⁡,*t*_(K). An alternative modification of the Norris and Landzberg (NL) model was introduced by Pan et al. [31]. It accounts for the dwell time *t*_0_ and *t*_*t*_ instead of the cycle frequency as it affects the creep damage the most. The Pan model (modified NL model) can be expressed as follows:

(11) AFPan=N0Nt=(ΔTtΔT0)2.65(ttt0)0.136×exp⁡{2185(1Tmax⁡,0−1Tmax⁡,t)},

where *N*_0_ and *N*_*t*_ represent the numbers of cycles to failure at a service condition and a test condition, respectively.

## 4. Results and Discussions

### 4.1. Failure Analysis and Microstructure Evolution

**Figure 4:**
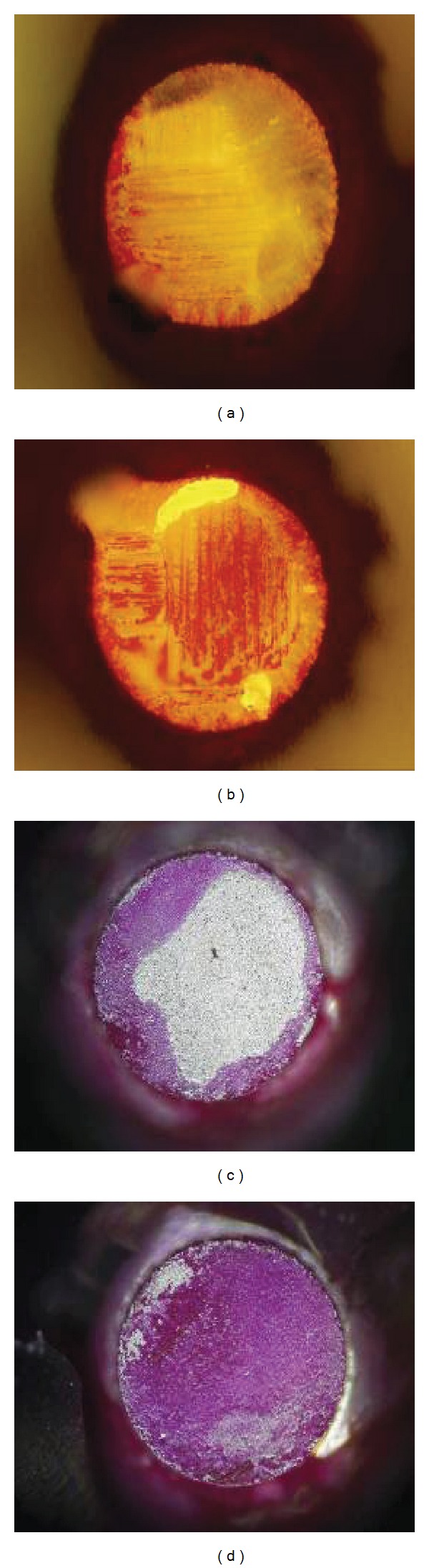
Optical images (200x) of dye penetration and dye rate. (a) 45%, (b) 100%, (c) 50% under polarized light, (d) 100% under polarized light.

**Figure 5:**
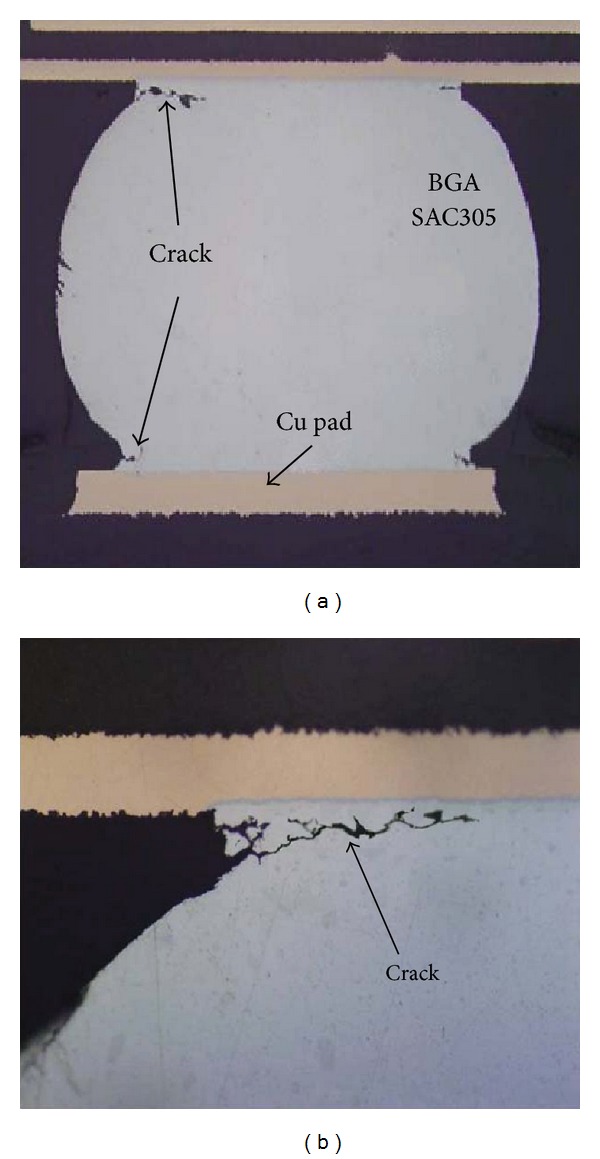
Crack image after 2500 thermal cycles. (a) A complete solder joint and (b) a corner of BGA side.

Figure 4 shows the dye and pry images of the BGA solder joints under normal and polarized lights after 2500 cycles. The edge of every solder ball has been dyed seriously, and the dyed area of some solder joints reaches 100% in BGA side.  Figure 5 is cross section images of the solders after 2500 thermal cycles. Cracks occur firstly at the interface of solder and Cu pad and have a tendency to extend along with the intermetallic compounds (IMC) layer. This test shows that the fatigue fracture of lead-free solder joints under the thermal cycling test is creep fracture, where the crack initiation always occurs in external edges of solders. The cracks are more obvious at the four corners of solders and devices under the maximal load as shown in Figure 5(b).

**Figure 6:**
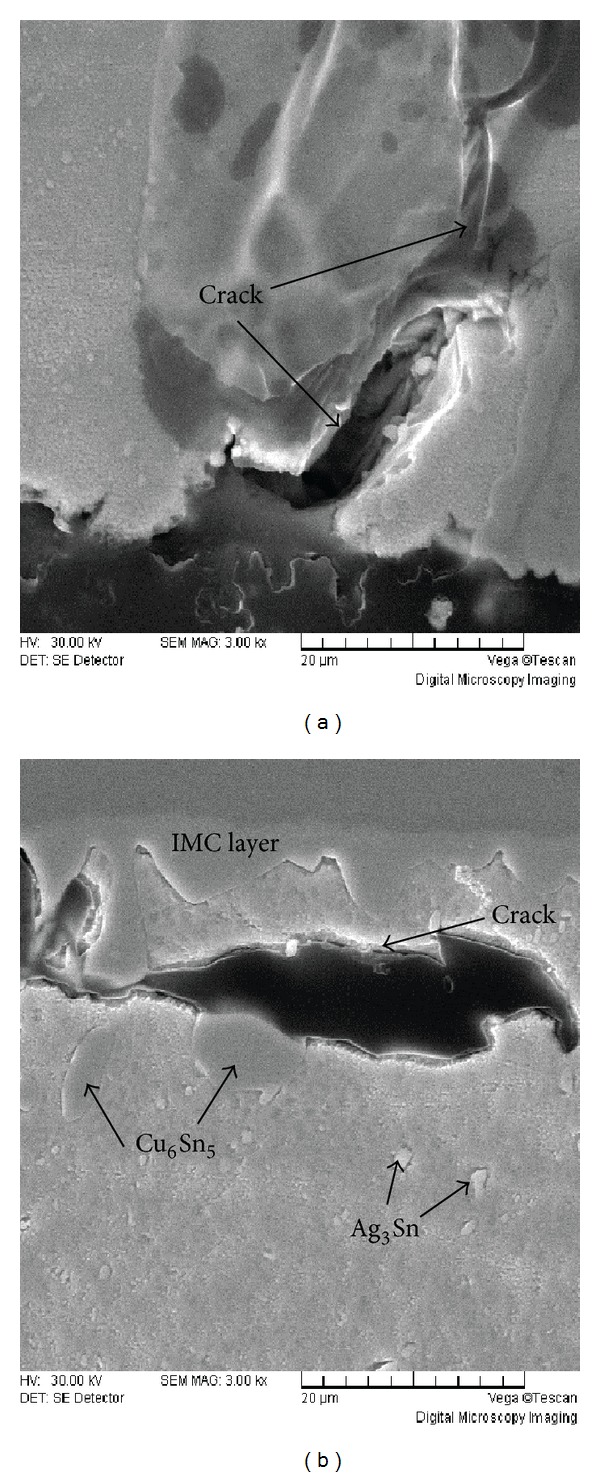
SEM image of the solder joint interface at the BGA side after 2500 thermal cycles.

Figures 6(a) and 6(b) show the cross section SEM images of solder joints after 2500 thermal cycles. The interfaces of the solder joints are mainly composed of scallop-type Cu_6_Sn_5_ after reflowing, and the mixtures of Ag_3_Cu and Cu_6_Sn_5_ form some white particles around IMC layer. With the increase of thermal cycles, the IMC layers of solder joints are gradually formed by Cu_6_Sn_5_ and Cu_3_Sn; then a layer of Cu_3_Sn grows up between Cu pad and Cu_6_Sn_5_. This is because the chemical reaction of atom Cu and Sn generates Cu_3_Sn and then Cu_3_Sn layer keeps growing with thermal cycles, which makes the scallop-type of IMC layer turn into flat-type. The area around the SnAgCu/Cu interface has become the weak point of the solder ball, because the increasing Cu_3_Sn layer has obvious fragility and low strength. This will cause crack initiation and propagation in the solders and further reduce the reliability of the solder joints.

### 4.2. Test Data Processing Based on Previous Methods

#### 4.2.1. Life Prediction Based on Engelmaier Model

Prior to the use of the Engelmaier fatigue model for life prediction of solder joints, the three parameters in (3) must be determined, where the creep fatigue is not considered in the Engelmaier fatigue model. According to the previous results [32], the fatigue ductility coefficient *ε*_*f*_ = 0.325 was used for a eutectic tin-lead solder. The fatigue ductility exponent* c* can be calculated by

(12) $$\begin{array}{c} c=-0.442-\left(6\times 10^{-4}\right)T_{\text{SJ}}+1.74\times 10^{-2}\ln\left(1+\frac{360}{t_{d}}\right), \end{array}$$

where *T*_SJ_ is a mean cyclic solder joint temperature during accelerated temp cycling (°C), *t*_*d*_ is a half cycle dwell time (minutes), and 360/*t*_*d*_ represents the cyclic frequency and 1 ≤ 360/*t*_*d*_ ≤ 1000 cycles/day [32]. The cyclic fatigue damage Δ*γ* for a lead-free solder can be written as follows:

(13) $$\begin{array}{c} \Delta \gamma =F\left(\frac{L_{D}}{h}\right)\Delta \alpha \Delta T_{e}=F\left(\frac{L_{D}}{h}\right)\left(\alpha_{s}\Delta T_{s}-\alpha_{c}\Delta T_{c}\right), \end{array}$$

where *h* is the nominal height of the solder joint, 2*L*_*D*_ is the maximum distance between the solder joints of component, *α*_*s*_, *α*_*c*_, Δ*T*_*s*_, and Δ*T*_*c*_ are coefficients of thermal expansions (CTE) and the cyclic temperatures of substrate and component, respectively, and Δ*α* represents the absolute difference between CTE of component and substrate (CTE mismatch). *F* is an empirical correction factor under idealized assumptions. The value of *F* varies from 0.5 to 1.5 and is set to 1 for lead-free solder SnAgCu [33]. It is assumed that temperature variation is relatively slow, and the temperature is evenly distributed in the component and the substrate. As a result, Δ*T*_*s*_ equals *c* = −0.57 and further results in Δ*T*_*e*_ = Δ*T*_*s*_ = Δ*T*_*c*_ [24].

The parameters of the Engelmaier fatigue model for (3), (12), and (13) are listed in Table 1. The zero stress reference temperature *T*_0_ has no effect on the analysis result of cyclical loading. With the ambient temperature *T*_0_ = 25°C, based on the defined package assembly architecture in Table 1, the cycles to failure caused by the thermal cycle are calculated by using (2) and (3). The parameter *c* = −0.401 is then determined. The fatigue lives for BGA and FBGA solder joints at 50% failure probability are *N*_*f*_(50%)_BGA_ = 2146.2 and *N*_*f*_(50%)_FBGA_ = 2375, respectively [34].

**Figure 7:**
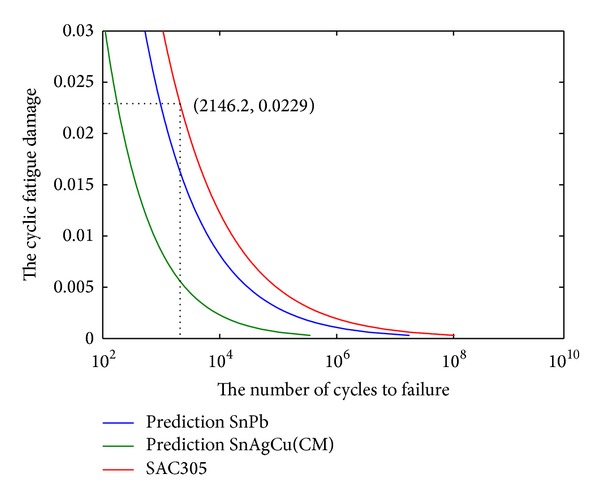
The predictions of Engelmaier fatigue model.

According to the previous studies, the fatigue ductility exponent* c* is within the range from −0.5 to −0.7 for common engineering metals [26]. For SnPb solder, *c* = −0.442, and SnAgCu solder, *c* = −0.57. The Engelmaier fatigue prediction method is applied. A comparison of predicted fatigue life between the two exponents with the fatigue ductility exponent in this paper that *c* = −0.401 is depicted in Figure 7. From this figure, we can conclude that the cyclic fatigue damage changes with the number of cycles to failure at different fatigue ductility exponents.

The result shown in Figure 7 indicates that the cyclic fatigue damage Δ*γ* (the cyclic plastic strain range) is decreasing as the test cycles at a different fatigue ductility exponent are increasing. The change of the fatigue ductility exponent *c* has a great influence on the mean number of cycles to failure at a fixed Δ*γ*.

#### 4.2.2. Life Prediction Based on Type-I Censored Data

Solder joint fails when its dyed area reaches up to 30% or the number of failed solder joints is no less than 10 percent of the total number at observation time *c*_*k*_. The connection structure is considered as failure, which means *δ*_*k*_ = 1. According to Section 3, the collected type-I interval censored data are tabulated in Table 2. They are the inputs from (6) to (9) for calculating the scale parameter *η* and shape parameter *m* of the Weibull distribution. Based on these data, we have obtained that *η*_1_ = 3104.5, *m*_1_ = 1.1 for BGA and *η*_2_ = 3185, *m*_2_ = 1.44 for FBGA, respectively.

**Figure 8:**
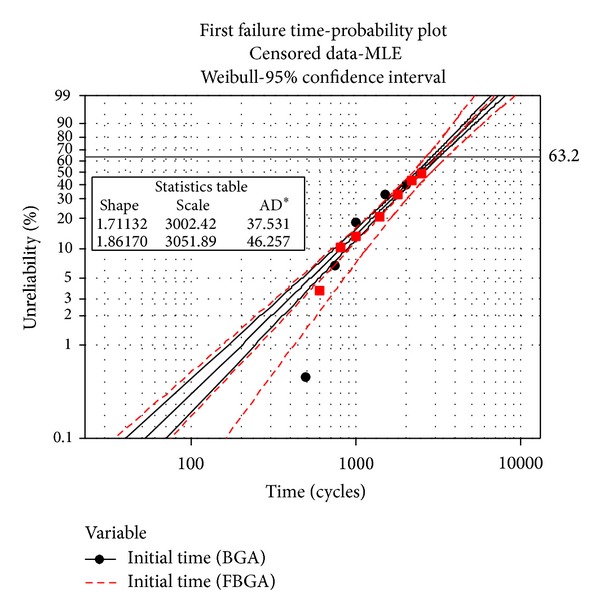
The probability plot of cycles to failure.

**Figure 9:**
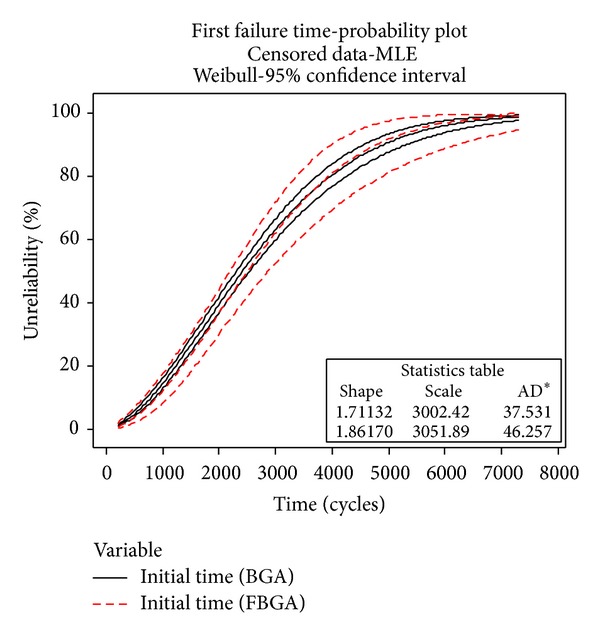
The cumulative failure plot of cycles to failure.

In the case of Weibull distribution, Minitab statistical software is used to analyze the experiment data (for BGA and FBGA) and calculate *m* using a built-in MLE method. The input data of Minitab are collected and settled in Table 3 [35]. The slope of the line, the *η*, the characteristic life, and the point at which 63.2% of the items in the data set have failed can be calculated. At last, with the aid of Minitab internal functions, the probability plot of cycles to failure is obtained by using the MLE method and it is shown in Figure 8, where the *x*-axis is the observation time and the *y*-axis is the cumulative failure probability.  Figure 9 shows the cumulative failure plot of cycles to failure of the BGA and FBGA solder joints on 95% confidence interval; the scale parameter and the shape parameterof BGA solder are *η*_1_′ = 3002.4 and *m*_1_′ = 1.7113, respectively. For FBGA, these two parameters are *η*_2_′ = 3052 and *m*_2_′ = 1.8617.

**Figure 10:**
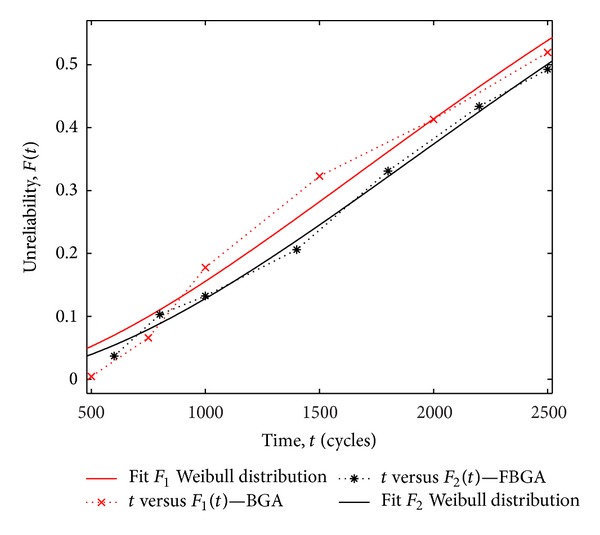
The fitting curve of Weibull distribution (the BGA and the FBGA).

**Figure 11:**
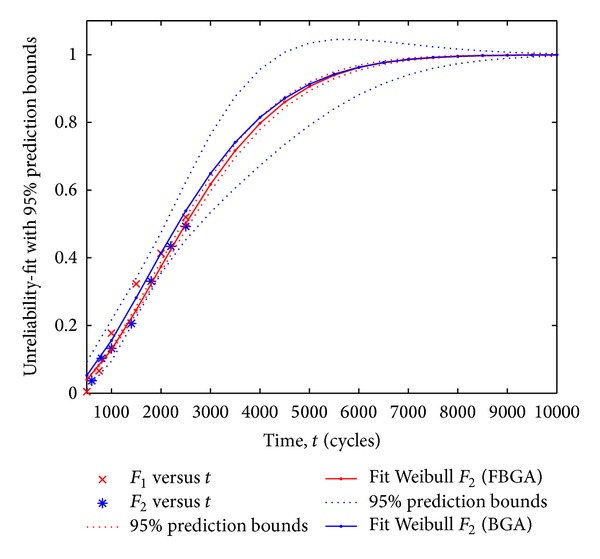
Analysis of two-parameter Weibull distribution fitting (the BGA and the FBGA).

**Figure 12:**
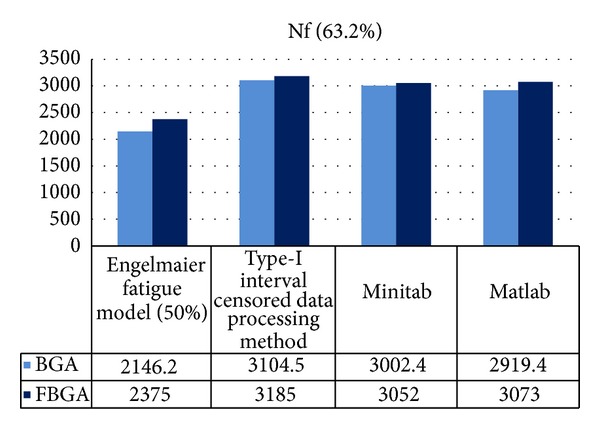
A comparison of the BGA and the FBGA solders fatigue life obtained using different methods.

At the same time, Matlab curve fitting toolbox is used to analyze the data from this experiment. The fitted curves of the Weibull distribution for the BGA and FBGA solder joints are shown in Figure 10, and the 95% prediction bounds of fitted Weibull distribution are plotted in Figure 11. The shape parameters and scale parameters of the BGA and FBGA *m*_1_′′, *η*_1_′′, *m*_2_′′, and *η*_2_′′, as well as the three other parameters, the sum of squares due to an error of the fitted curve (SSE), the *R*-square coefficient of determination, and the root mean square error (RMSE), which characterize the goodness of fit, are obtained and listed in Table 4. The comparison of the fatigue life predicted by using Engelmaier fatigue model and type-I interval censored data processing method with the ones obtained for SAC305 BGA and FBGA solder joints using Matlab and Minitab is shown in Figure 12.

#### 4.2.3. Acceleration Factor Estimation

Since the Coffin-Manson is more conservative than the NL model and the Pan model in terms of estimating the acceleration factor, the activation energy of the NL model is calibrated for SnPb solder alloys. In this paper, for SnAgCu solder alloys, the Pan model is used to calculate the acceleration factor for predicting field life. The test condition and parameters of (11) are listed in Table 5. Then the acceleration factor under the Pan model is obtained:

(14) AFPan=N0Nt=(ΔTtΔT0)2.65(ttt0)0.136×exp⁡{2185(1Tmax⁡,0−1Tmax⁡,t)}=(16560)2.65(10.25)0.136exp⁡{2185(1353−1398)}=35.5.

It means that the Pan acceleration factor of this thermal cycling test is approximated to be 35.5.

### 4.3. Discussions

The failure analysis and microstructure evolution of the solder joints in this test show that the fatigue fracture of the lead-free solder joints under thermal cycling test is creep fracture. The crack initiation always occurs in external edges of solders. Cracks are mainly grown at the interface of solder and Cu pad and have a tendency to extend along with the intermetallic compounds (IMC) layer. With the increase of thermal cycles, the IMC layers of solder joints are gradually divided into two layers, namely, Cu_6_Sn_5_ layer and Cu_3_Sn layer. Because of the obvious fragility caused by the increase of IMC layer and holes in solders, SnAgCu/Cu interface becomes the weak point of the whole solders with a low strength.

Figure 12 compares the predicted life of the BGA and FBGA lead-free solder joints obtained using the modified Engelmaier fatigue model and the Type-I interval censored data processing method with the results obtained using Matlab curve fitting and Minitab statistical software. For the BGA solders, the median life obtained by using the modified Engelmaier fatigue model is 2146.2 cycles, the life got from type-I interval censored data processing method is 3002.4 cycles, and the characteristic life calculated by Matlab and Minitab software is 2919.4 cycles. For FBGA solder joints, these four predictions of life are 2375, 3185, 3052, and 3073 cycles, respectively.

The basic theory of Matlab curve fitting toolbox is Powell's Dogleg Method, which is a hybrid algorithm originating from the Levenberg-Marquardt method and the Newton-steepest descent method. Powell's Dogleg Method is an excellent algorithm for nonlinear curve fitting, and the fitting results have high credibility [36]. If the parameter of SSE is close to 0, it is indicated that the model fitting is good, and the data prediction is credible. The coefficient of determination, *R*-square, characterizes the quality of the curve fitting through data changing. If *R*-square is close to 1, it means that the variable of the equation has strong ability to explain the function and this model fits the data well. Minitab statistical software, which is based on the built-in maximum likelihood estimation (MLE) method, is used to estimate the Weibull parameters. It adopts similar principle with type-I interval censored data processing method which is introduced in Section 3. Matlab and Minitab are used to analyze the experimental data, respectively. The results obtained using those two prediction methods can represent the experiment condition approximately.

From Table 4, we know that for the BGA, SSE_1_ = 0.005943 and *R*-square_1_ = 0.9709 and for the FBGA, SEE_2_ = 0.000957 and *R*-square_2_ = 0.9947. Both SSEs are close to 0 and both *R*-squares are close to 1, which mean the curve fitting by Matlab is effective and accurate. All of these predictions match the experimental results with a high confidence, and the methods used in this paper are applicable for lead-free solder joint life prediction. In Section 4.2, the shape and scale parameters for the BGA using Matlab curve fitting toolbox are *m* = 1.658 and *η* = 2919.4, respectively. The acceleration factor of this test is nearly 35.5, and the number of cycles to failure under the normal operating condition can be estimated easily. Using the equations in Section 3, the reliability characteristics of SAC305 lead-free solder joint including MTTF, *R*(*t*_0_), and *t*_*R*_ can be calculated.

Using the modified Engelmaier fatigue model to predict solder joint fatigue life (cycles to failure) is only associated with the total shear strain and the modified fatigue ductility exponent, where these two parameters can be obtained easily. Therefore, without considering other effect factors, this model is an effective method for the solder joint lifetime prediction. The results obtained in this paper show that the type-I interval censored data processing method which is applicable for censored data generated from accelerated life tests is an effective method to predict fatigue life of lead-free solder joints.

## 5. Conclusions

The modified Engelmaier fatigue model is a reasonable and effective method for estimating the fatigue life of solder interconnects. However, this model is more conservative than other models. Through the analyses of the censored lifetime data of thermal cycling tests, it is found that the type-I censored data processing method is an effective method to predict the life of solder joint. The comparison study carried out using Minitab indicates that the type-I interval censored data processing method can predict the failure life of lead-free solder joint effectively. This study shows that the fatigue fracture of lead-free solder joint under thermal cycling test is creep fracture, and the crack initiation always occurs at the interface of solder and Cu pad. The IMC layer is gradually divided into Cu_6_Sn_5_ and Cu_3_Sn layers, and the area close to SnAgCu/Cu interface has become the weak point of the whole solder because of the fragility introduced by the increase of IMC layer and holes in solders. Being the weaknesses of microelectronic packaging, the reliability of solder joint interconnections affects the reliability of the entire structure. The prediction of the solder joint life can offer suggestions for regular maintenance, replacement of substructure, and subsequent processing of electronics.

## Figures and Tables

**Table 1:** The parameters for the Engelmaier fatigue model.

Parameters	Explanation	BGA (SAC305)	FBGA (SAC305)
*L*_*D*_	2*L*_*D*_ is the maximum distance between the solder joints of component (mm)	23.3345	7.7666
*h*	The nominal height of the solder joint (mm)	0.42	0.25
Δ*T*	Cyclic temperature Δ*T*_*e*_ = Δ*T*_*s*_ = Δ*T*_*c*_ (°C)	165	165
*F*	Empirical correction factor ([0.5, 1.5])	1	1
*ε*_*f*_	Fatigue ductility coefficient	0.325	0.325
*T*_0_	Zero stress reference temperature (°C)	25	25
*t*_*d*_	A half cycle dwell time (mins)	15	15

**Table 2:** Type-I interval censored data of ATC.

BGA	*c*_*i*_	0	250	500	750	1000	1500	2000	2500
	*δ*_*i*_	0	0	1	0	0	1	1	0
	*c*_*i*_	0	250	500	750	1000	1500	2000	2500
	*δ*_*i*_	0	0	0	0	1	0	0	1

FBGA	*c*_*i*_	300	600	800	1000	1400	1800	2200	2500
	*δ*_*i*_	0	0	0	1	0	0	1	0
	*c*_*i*_	300	600	800	1000	1400	1800	2200	2500
	*δ*_*i*_	0	0	0	0	1	0	0	1

**Table 3:** The input data of Minitab.

BGA				FBGA			
Initial time (cycles)	Inspection time (cycles)	Failure frequency	Cumulative failure prob. (%)	Initial time (cycles)	Inspection time (cycles)	Failure frequency	Cumulative failure prob. (%)
0	250	0	0	0	300	0	0
250	500	5	0	300	600	5	3.68
500	750	71	0.43	600	800	9	10.29
750	1000	129	6.58	800	1000	4	13.24
1000	1500	168	17.75	1000	1400	10	20.59
1500	2000	74	32.29	1400	1800	17	33.09
2000	2500	123	41.3	1800	2200	14	43.38
2500	∗	556	51.95	2200	2500	8	49.26
				2500	∗	69	

**Table 4:** The results obtained by using Matlab.

Parameters	*m*′′	*η*′′	SSE	*R*-square	RMSE
BGA	1.658 (1.087, 2.229)	2919	0.0059	0.9709	0.0385
FBGA	1.769 (1.762, 1.776)	3073	0.00096	0.9947	0.0126

**Table 5:** Parameters for acceleration factor calculations.

Parameters	*T*_max⁡_ (°C)	*T*_min⁡_ (°C)	Δ*T* (°C)	Cycles per hour	Dwell time (mins)
Test condition	125	−40	165	1	15
Field condition	80	20	60	0.25	360
